# Comparison of intermittent and continuous renal replacement therapy for sepsis-associated AKI: a retrospective analysis of the Japanese ICU database

**DOI:** 10.1186/s40981-025-00787-8

**Published:** 2025-04-28

**Authors:** Hiromu Okano, Hiroshi Okamoto, Haruna Tanaka, Ryota Sakurai, Tsutomu Yamazaki

**Affiliations:** 1 Department of Critical Care Medicine, St. Luke’s International Hospital, 9-1 Akashi-cho, Chuo-ku, Tokyo, 104-8560 Japan; 2 Department of Social Medical Sciences, Graduate School of Medicine, International University of Health and Welfare, 4-1-26 Akasaka, Minato City, Tokyo, 107-8402 Japan; 3 Department of Biostatistics, Graduate School of Medicine, The University of Tokyo, 7-3-1 Bunkyo-ku, Tokyo, 113-8655 Japan

**Keywords:** Intermittent renal replacement therapy, Continuous renal replacement therapy, Sepsis-associated acute kidney injury

## Abstract

**Introduction:**

While both intermittent renal replacement therapy (IRRT) and continuous renal replacement therapy (CRRT) are used to treat sepsis-associated acute kidney injury (S-AKI), their comparative effectiveness remains unclear. We compared the outcomes between these modalities in patients with S-AKI.

**Methods:**

Data from the Japanese Intensive Care Patient Database (JIPAD) was used for this multi-center retrospective cohort study. Adult patients with S-AKI who received either IRRT or CRRT between 2015 and 2021 were included. The primary outcome was in-hospital mortality. We compared IRRT and CRRT using one-to-three propensity score matching analysis. A subgroup analysis was performed in patients with septic shock.

**Results:**

Of the 756 patients analyzed, 79 received IRRT, and 677 received CRRT. After propensity score matching, baseline characteristics were well-balanced between groups. In-hospital mortality showed no significant difference between IRRT and CRRT (48.6% vs. 38.0%; risk difference − 10.6%; 95% CI − 23.0 to 2.9; *P* = 0.11). In patients with septic shock, in-hospital mortality was also not different between groups (52.6% vs. 40.4%; risk difference − 12.2%; 95% CI − 28.8 to 3.7; *P* = 0.10).

**Conclusion:**

IRRT and CRRT may be similar in-hospital mortality in patients with S-AKI. Further studies are warranted to determine the most effective renal replacement modality for this patient population.

## Background

Sepsis remains a leading cause of death in intensive care units worldwide. Acute kidney injury (AKI) complicates approximately 60% of sepsis cases, and sepsis-associated AKI (S-AKI) significantly increases mortality [1, 2]. Among patients requiring renal replacement therapy (RRT) for S-AKI, mortality rates reach approximately 50% [3]. Therefore, specific treatment for S-AKI is important. To date, however, the optimal RRT modality for S-AKI remains unclear.

The two main RRT modalities for S-AKI are intermittent renal replacement therapy (IRRT) and continuous renal replacement therapy (CRRT). CRRT provides continuous blood purification at a lower intensity, while IRRT delivers higher-intensity treatment intermittently. Although CRRT has traditionally been preferred for hemodynamically unstable patients [4, 5].

Current clinical guidelines, including the 2021 Surviving Sepsis Campaign and the 2020 Japanese Guidelines, do not specify a preferred RRT modality for S-AKI [6, 7]. This uncertainty stems from randomized trials that included critically ill patients with various causes of AKI, not specifically S-AKI [8–12]. Moreover, RRT practices and outcomes vary considerably across countries [13, 14].

In Japan the choice between CRRT and IRRT is often influenced by institutional practices rather than specific clinical guidelines, and the effects of this choice on patient outcomes in sepsis-associated AKI remain uninvestigated. The optimal RRT modality for S-AKI remains unclear. Therefore, using the Japanese Intensive Care Patient Database (JIPAD) [15], we compared in-hospital mortality between IRRT and CRRT in patients with S-AKI.

## Methods

### Study design, population, and setting

We conducted a multicenter retrospective cohort study using data from the JIPAD, the largest intensive care database in Japan [15]. As of June 2023, the database included 250,672 individuals from 83 facilities. This study follows the Strengthening the Reporting of Observational Studies in Epidemiology (STROBE) statement [16].

The protocol for this research project was approved by a suitable constituted Institutional Ethics Committee (Committee of International University School of Health and Welfare Graduate School, Approval No. 23-Im-058, and the Ethics Committee of St. Luke’s International Hospital, Approval No. 23-R049), and it conforms to the provision of the Declaration of Helsinki.

### Participants

This study focused on S-AKI, a subtype of AKI primarily caused by sepsis [17], including patients diagnosed with sepsis or septic shock who were considered to have developed S-AKI and required renal replacement therapy between 2015 and 2021. The exclusion criteria were as follows: age < 18 years, maintenance dialysis, cardiac arrest on admission, intensive care unit (ICU) readmissions, procedural admissions, transfer to the coronary care unit (CCU) or other ICUs/hospitals, receiving both IRRT and CRRT, unknown use of antihypertensive medications, missing height/weight data, or ICU stay < 24 h.

### Exposure and outcomes

Patients were categorized into the IRRT or CRRT groups based on treatment during their ICU stay. The primary outcome was in-hospital mortality. Secondary outcomes were hospital and ICU length of stay.

### Variables

The following data were extracted from the JIPAD: year of admission, age, sex, weight, height, ICU/hospital stay duration, chronic comorbidities (including congestive heart failure, respiratory failure, liver failure, cirrhosis, immunosuppressants use, lymphoma, acute leukemia, and metastatic cancer), ICU admission reason (planned/emergency), surgery type (planned/emergency), diagnostic codes, Acute Physiologic and Chronic Health Evaluation (APACHE) II and III scores, Simplified Acute Physiology Score II (SAPS II), and Sequential Organ Failure Assessment (SOFA) score. Data on mechanical ventilation, acute kidney injury, and the use of dopamine, noradrenaline, dobutamine, and adrenaline were also obtained. Body mass index (BMI) was calculated and categorized per the World Health Organization definitions.

### Statistical analysis

Propensity score analysis was used to adjust for baseline differences between the CRRT and IRRT groups. Propensity scores were calculated using logistic regression with variables such as age, sex, BMI, comorbidities, planned surgery, surgical categories, APACHE II and III scores, SAPS II score, SOFA score, mechanical ventilation, AKI, and catecholamine levels. Propensity score analysis was used to adjust for baseline differences between the CRRT and IRRT group. The discriminatory ability of the logistic regression model to differentiate between patients receiving CRRT and IRRT was evaluated using the C-statistic. A C-statistic value between 0.6 and 0.9 was considered optimal for achieving sufficient discrimination without overfitting or insufficient separation between treatment groups [18]. Given the imbalance in group sizes, we used 1:3 nearest-neighbor matching without replacement to maximize comparability while preserving statistical power. It was performed based on estimated propensity scores, with a caliper width of 20% of the standard deviation of the logit-transformed propensity scores. Baseline characteristics balance was assessed using absolute standardized differences, with values < 10% considered balanced. Categorical variables were expressed as numbers and percentages, and continuous variables were expressed as medians with interquartile ranges (IQRs).

Risk differences and 95% confidence intervals (CIs) for in-hospital mortality were calculated after matching. The chi-square test was used for comparing groups. Median and IQR were calculated for hospital and ICU length of stay. The Wilcoxon rank-sum test was used to compare two groups. Two-sided *P* values < 0.05 were considered statistically significant. Regarding the sample-size calculation, based on observed mortality rates (50.0% in the CRRT group and 31.1% in the IRRT group) [19], with a significance level (α) of 0.05 and power of 80%, 209 patients in total (157 in the CRRT group and 52 in the IRRT group, at a 3:1 ratio) were required to detect a statistically significant difference in mortality between the two dialysis modalities. Analyses were conducted using SPSS software (version 29.0; SPSS Inc., Chicago, IL, USA) and R version 4.0.2 (R Foundation for Statistical Computing, Vienna, Austria).

### Subgroup analyses

We performed subgroup analyses in patients with septic shock and S-AKI to examine the effects of specific interventions and conditions.

### Sensitivity analyses

We conducted a sensitivity analysis using inverse probability of treatment weighting (IPTW) to assess the robustness of findings for patients with S-AKI who received IRRT or CRRT, following the same methodology as the main analysis. Additionally, we estimated the average treatment effect (ATE) to further validate the robustness of our findings, using IPTW to account for potential confounding variables.

## Results

### Participant selection

**Fig. 1:**
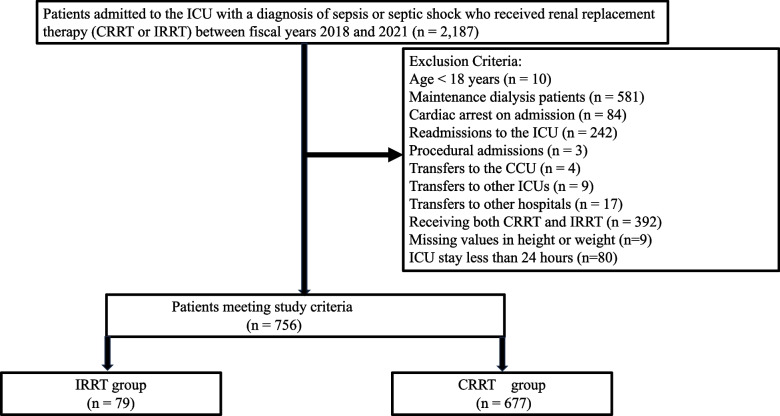
Patient selection flowchart for examining renal replacement therapy (RRT) in patients admitted to the ICU with sepsis-associated AKI between 2015 and 2021. The initial cohort included 2577 patients. After applying the exclusion criteria, 756 patients met the study criteria. These patients were divided into two groups: 677 received CRRT and 79 received IRRT. AKI, acute kidney injury; CRRT, continuous renal replacement therapy; IRRT, intermittent renal replacement therapy; ICU, intensive care unit

**Fig. 2:**
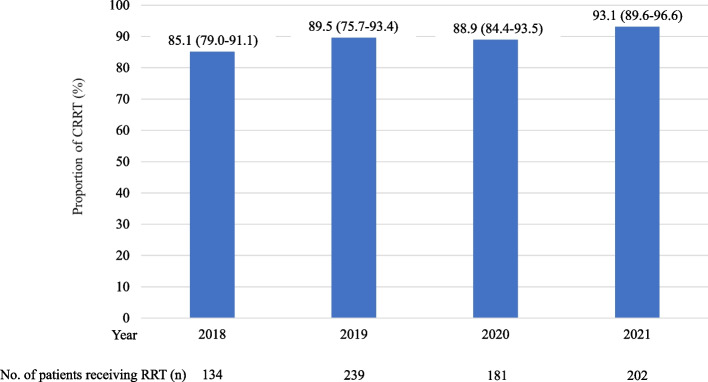
Annual distribution of RRT and the percentage of CRRT performed for sepsis-associated AKI, shown as percentages with 95% CIs. The blue bars indicate the percentage of CRRT. CI, confidence interval; CRRT, continuous renal replacement therapy; RRT, renal replacement therapy

Figure 1 summarizes the participant selection process. Among 2557 patients with S-AKI who received IRRT or CRRT registered in the JIPAD between 2015 and 2021, 756 were included in the analysis. Of these, 677 patients (89.6%) received CRRT, while 79 (10.4%) received IRRT. The annual results are shown in Fig. 2, with CRRT performed in 85.1% to 93.1% of the cases.

### Participant characteristics

Table 1 displays the baseline characteristics of patients before propensity score matching, revealing significant differences between the groups. Standardized mean differences (SMDs) exceeded 0.1 for the body mass index (BMI), ICU admission reasons, heart failure, leukemia or lymphoma, APACHE II and III scores, SAPS II score, SOFA score, mechanical ventilation within the first 24 h, AKI within the first 24 h, and use of catecholamines, noradrenaline, dobutamine, and adrenaline. SMDs used for matching were listed. The C-statistic calculated for propensity score matching was 0.69, which fell within the optimal range. Table 2 presents the results after 1:3 propensity score matching, where 79 IRRT patients and 212 CRRT patients were analyzed. Only cancers with metastases showed an absolute SMD > 0.1 (SMD = 0.11), indicating appropriate matching.

**Table 1:** Baseline characteristics of patients before propensity score matching

	IRRT (*n* = 79)	CRRT (*n* = 677)	SMD
Age	72 (63.5,79.5)	72 (63,80)	0.02
Male (%)	45 (57.0%)	400 (59.1%)	0.04
BMI	22.07 (19.5,25.7)	22.5 (19.94,25.66)	0.17
Reason for ICU admission (%)			
Transfer from ward	36(45.6%)	243(35.89%)	
Transfer from emergency room	4 (5.1%)	81(11.96%)	
Elective surgery	26 (32.9%)	275(40.62%)	
Urgent surgery	5 (6.3%)	96 (14.18%)	0.32
Comorbidities (%)			
Heart failure	0 (0.0%)	12 (1.77%)	0.14
Respiratory failure	1 (1.3%)	7 (1.03%)	0.02
Cirrhosis	4 (5.1%)	28 (4.14%)	0.04
Liver failure	2 (2.5%)	14 (2.07%)	0.03
Use of immunosuppressants	12 (15.2%)	111 (16.40%)	0.03
Acquired immune deficiency syndrome	0 (0.0%)	2 (0.30%)	0.06
Acute leukemia or lymphoma	0 (0.0%)	17 (2.51%)	0.17
Cancer with metastases	3 (3.8%)	29 (4.28%)	0.03
Lymphoma	4 (5.1%)	45 (6.65%)	0.07
APACHE2	27 (23,32)	28 (23,34)	0.18
APACHE3	103 (91,120)	107 (89,132)	0.21
SAPS2	57 (45.5,69.5)	61 (50,74)	0.27
SOFA	12(9,14)	12(10,15)	0.26
Mechanical ventilation during the first 24 h (%)	38 (48.1%)	470 (69.4%)	0.43
AKI during the first 24 h (%)	34 (43.0%)	235 (34.7%)	0.17
Catecholamine use	56 (70.9%)	613 (90.6%)	0.43
Dopamine	4 (5.1%)	67 (9.9%)	
Noradrenaline	56 (70.9%)	598 (88.3%)	
Dobutamine	11 (13.9%)	163 (24.1%)	
Adrenaline	2 (2.5%)	93 (13.7%)	

Values are presented as number (%) or median (Q1, Q3)

*AKI* acute kidney injury, *APACHE* Acute Physiologic and Chronic Health Evaluation, *BMI* body mass index, *CRRT* continuous renal replacement therapy, *ICU* intensive care unit, *IRRT* intermittent renal replacement therapy

**Table 2:** Baseline characteristics of patients after propensity score matching by logit (1:3)

	IRRT (*n* = 79)	CRRT (*n* = 212)	SMD
Age	72 (63.5,79.5)	71 (62,8)	0.04
Male (%)	45 (56.96%)	122 (57.6%)	0.009
BMI	22.1 (19.5,25.7)	22.8 (19.7,25.6)	0.09
Reason for ICU admission (%)			
Transfer from ward	36 (45.6%)	80 (37.7%)	
Transfer from emergency room	4 (5.1%)	12 (5.7%)	
Elective surgery	26 (32.9%)	99 (46.7%)	
Urgent surgery	5 (6.3%)	12 (5.7%)	0.05
Comorbidities (%)			
Heart failure	0 (0.0%)	0 (0.0%)	0
Respiratory failure	1 (1.3%)	3 (1.4%)	0
Cirrhosis	4 (5.1%)	11 (5.2%)	0.02
Liver failure	2 (2.5%)	5 (2.4%)	0.03
Use of immunosuppressants	12 (15.2%)	36 (17.0%)	0.06
Acquired immune deficiency syndrome	0 (0.0%)	0 (0.0%)	0
Acute leukemia or lymphoma	0 (0.0%)	0 (0.0%)	0
Cancer with metastases	3 (3.8%)	12 (5.7%)	0.11
Lymphoma	4 (5.1%)	7 (3.3%)	0.10
APACHE2	27 (23,32)	28 (22,34)	0.006
APACHE3	103 (91,120)	107 (88.0,130.3)	0.002
SAPS2	57 (45.5,69.5)	58 (48,70)	0.005
SOFA	12 (9,14)	11.5 (9,14)	0.07
Mechanical ventilation during the first 24 h (%)	38 (48.10%)	123 (58.1%)	0.08
AKI during the first 24 h (%)	34 (43.0%)	88 (41.5%)	0.03
Catecholamine use	56 (70.9%)	161 (75.9%)	0.07
Dopamine	4 (0.6%)	15 (7.1%)	
Noradrenaline	56 (8.3%)	158 (74.5%)	
Dobutamine	11 (1.6%)	39 (18.4%)	
Adrenaline	2 (0.3%)	24 (11.3%)	

Values are presented as number (%) or median (Q1, Q3)

*AKI* acute kidney injury, *APACHE* Acute Physiologic and Chronic Health Evaluation, *BMI* body mass index, *CRRT* continuous renal replacement therapy, *ICU* intensive care unit, *IRRT* intermittent renal replacement therapy

### Clinical outcome

There were no significant differences in in-hospital mortality between the IRRT and CRRT groups (38.0% vs. 48.6%; risk difference − 10.6%; 95% CI − 23.0 to 2.9; *P* = 0.11) (Table 3). There was a significant difference in hospital length of stay, with the IRRT group having a longer median stay of 42 days compared to 31 days in the CRRT group (*P* = 0.01). However, ICU length of stay was similar between the groups (IRRT 5 days vs. CRRT; 6 days; *P* = 0.30).

**Table 3:** Outcomes after propensity score matching by logit (1:3)

Outcomes	IRRT (*n* = 79)	CRRT (*n* = 212)	Risk difference (95% CI)	*P* value
In-hospital mortality	30 (38.0%)	103 (48.6%)	− 10.6 (− 23.0, 2.9)	0.11
Hospital length of stay (days)	42 (23.5,65.5)	31 (15,61)	–	0.01
ICU length of stay (days)	5(3,9)	6 (3,11)	–	0.30

Values are presented as number (%) or median (Q1, Q3)

*ICU* intensive care unit

### Subgroup analyses

Patients with septic shock showed no significant difference in in-hospital mortality between groups (40.4% vs. 52.6%; risk difference − 12.2%; 95% CI − 28.8 to 3.7; *P* = 0.10) (Table 4).

**Table 4:** Subgroup outcomes after propensity score matching (1:3)

Outcomes	IRRT (*n* = 57)	CRRT (*n* = 156)	Risk difference (95% CI)	*P* value
In-hospital mortality	23 (40.4%)	82 (52.6%)	− 12.2 (− 28.8, 3.7)	0.10
Hospital length of stay (days)	41 (23,60)	27 (12,55)	–	0.02
ICU length of stay (days)	6 (3,10)	6 (3,10)	–	0.90

Values are presented as number (%) or median (Q1, Q3)

*ICU* intensive care unit

### Sensitivity analyses

Sensitivity analysis using IPTW, estimating the average ATE, also indicated no significant difference in in-hospital mortality (38.0% vs. 49.6%; risk difference − 5.33%; 95% CI − 19.1 to 8.0; *P* = 0.06) (Table 5).

**Table 5:** Outcomes after inverse probability weights (stabilized IPTW)

Outcomes	IRRT (*n* = 79)	CRRT (*n* = 667)	Risk difference (95% CI)	*P* value
In-hospital mortality	30 (38.0%)	336 (49.6%)	− 5.33 (− 19.1, 8.0)	0.06
Hospital length of stay (days)	42 (23.5,65.5)	30 (13,56)	–	< 0.001
ICU length of stay (days)	5 (3,9)	6 (3,11)	–	0.30

Values are presented as number (%) or median (Q1, Q3)

*ICU* intensive care unit

## Discussion

In this large-scale multicenter retrospective cohort study using JIPAD, we compared the mortality outcomes between CRRT and IRRT in patients with sepsis or septic shock who developed S-AKI. In-hospital mortality was comparable between the two RRT modalities.

Previous studies examining RRT modalities in critically ill patients have shown conflicting results. Iwagami et al. [19] analyzed a Japanese health insurance claims database and reported lower mortality associated with IRRT compared to that associated with CRRT. However, their study was limited by potential selection bias and unmatched disease severity between groups. Our study addressed these limitations through propensity score matching and focused specifically on patients with S-AKI. Furthermore, considering that in Japan the choice of RRT modality is often determined by institutional or physician preference rather than patient-specific factors, our study investigated whether this choice affected patient outcomes when illness severity was balanced via matching.

Our findings challenge the common preference for CRRT in septic patients. While CRRT is often selected for hemodynamically unstable patients [19, 20], we found no significant mortality difference between the two treatment modalities. This finding aligns with a recent study by Nogi et al. [21], who demonstrated that IRRT can effectively manage metabolic derangements without compromising hemodynamic stability. There was no significant difference in mortality in the current study, and this may indicate that both CRRT and IRRT can be appropriately applied in ICU settings by trained staff. Moreover, factors such as the timing of RRT initiation and overall sepsis management may play a more critical role than modality alone. This may reflect the high standards of care in JIPAD-participating ICUs, where both CRRT and IRRT are implemented under appropriate clinical supervision, potentially minimizing differences in treatment outcomes.

Current international guidelines, including the 2021 Surviving Sepsis Campaign, do not recommend one RRT modality over another, citing insufficient evidence [6, 22]. Our results support this position and suggest that RRT selection should be individualized rather than defaulting to CRRT for all septic patients.

With respect to the JIPAD data, CRRT was more often used in patients with higher illness severity scores and vasopressor use, suggesting hemodynamic instability. IRRT was more commonly used in hemodynamically stable patients. Although causal relationships cannot be inferred, these trends may assist clinicians in modality selection based on patient characteristics. Although the IRRT group comprised only 10.4% of patients, we performed matching and confirmed a good balance in baseline characteristics. Sensitivity analyses using IPTW and ATE estimation confirmed the robustness of our findings. Lastly, the small proportion of IRRT cases (approximately one-tenth of CRRT cases) may reflect institutional preferences rather than clinical indications.

While our findings suggest that both RRT modalities can be considered for S-AKI, larger randomized controlled trials are needed to establish their comparative effectiveness. Future studies should also focus on identifying optimal timing for IRRT initiation and discontinuation based on hemodynamic parameters.

Our study had some limitations. First, while we extracted data based on available variables for sepsis patients who received dialysis, S-AKI was insufficiently defined, and the definition of AKI used may not fully satisfy KDIGO guidelines [23]. Additionally, our analysis was limited to patients diagnosed with sepsis or septic shock who underwent renal replacement therapy. Second, the exact timing of dialysis initiation during ICU stay was not available, limiting the interpretation of our results. Third, we excluded patients who received treatment using both dialysis modalities because treatment sequence data were unavailable, although previous studies suggest that treatment sequence may affect patient outcomes [4, 19]. Fourth, despite robust findings in sensitivity analyses using IPTW, unmeasured confounding factors may still exist owing to the retrospective nature of this study. Fifth, the small proportion of IRRT cases (approximately one-tenth of CRRT cases) may reflect institutional preferences rather than clinical indications, potentially limiting the generalizability of our findings. Finally, the small proportion of IRRT cases (approximately one-tenth of CRRT cases) reflects current practice patterns in Japan but creates methodological challenges for comparative analysis. While our matching and sensitivity analyses attempted to address this imbalance, our findings should be interpreted with caution and confirmed in settings with more balanced utilization of both modalities.

## Conclusions

In patients with S-AKI, IRRT and CRRT showed similar in-hospital mortality. The choice of RRT modality should be individualized based on patient characteristics and institutional factors.

## Data Availability

The datasets generated in this study are available from the corresponding author upon request.

## Abbreviations

AKI: Acute kidney injury
APACHE: Acute Physiologic and Chronic Health Evaluation
ATE: Average Treatment Effect
BMI: Body Mass Index
CCU: Coronary care unit
CI: Confidence interval
CRRT: Continuous renal replacement therapy
ICU: Intensive care unit
IPTW: Inverse Probability of Treatment Weighting
IRRT: Intermittent renal replacement therapy
JIPAD: Japanese Intensive Care Patient Database
RRT: Renal replacement therapy
S-AKI: Sepsis-associated acute kidney injury
SAPS: Simplified Acute Physiology Score
SOFA: Sequential Organ Failure Assessment
STROBE: Strengthening the Reporting of Observational Studies in Epidemiology
